# Trends in cause of death among patients with renal cell carcinoma in the United States: a SEER-based study

**DOI:** 10.1186/s12889-023-15647-2

**Published:** 2023-04-26

**Authors:** Xiangpeng Zhan, Tao Chen, Ying Liu, Hao Wan, Xiaoqiang Liu, Xinxi Deng, Bin Fu, Jing Xiong

**Affiliations:** 1 Department of Urology, The First Affiliated Hospital of Nanchang University, Nanchang, Jiangxi Province China; 2 Department of Preventive Medicine, School of Public Health, Hubei University of Medicine, Shiyan, Hubei Province China; 3 Department of Urology, Jiu Jiang No.1 People’s Hospital, Jiujiang, Jiangxi Province China

**Keywords:** Renal cell carcinoma, Cause of death, Trend, SEER, Survival

## Abstract

**Background:**

Renal cell carcinoma (RCC) survival has improved due to recent developments in RCC treatment. Therefore, other co-morbid conditions may have a more critical role. This study aims to explore the common causes of death in patients with RCC to improve the management and survival of RCC.

**Method:**

We used the Surveillance, Epidemiology, and End Results (SEER) (1992–2018) database to get patients with RCC. We calculated the percentage of total deaths of six kinds of the cause of death (COD) and the cumulative incidence of death for each selected cause over survival time. The joinpoint regression was utilized to present the trend of mortality rate by COD.

**Results:**

We enrolled 107,683 cases with RCC. RCC was the leading cause of death in patients with RCC [25376(48.3%)], followed by cardiovascular diseases [9023(17.2%)], other cancers [8003 (15.2%)], other non-cancer diseases [4195 (8%)], non-disease cause [4023 (7.7%)], and respiratory diseases [1934 (3.6%)]. The proportion of patients who died of RCC decreased gradually over survival time, and this value decreased from 69.71% in 1992–1996 to 38.96% in 2012–2018. The non-RCC cause mortality rate showed an increasing trend, whereas a slight decrease was observed in RCC specific mortality rate. The distribution of such conditions varied across different patient populations.

**Conclusion:**

RCC was still the primary COD of patients with RCC. However, non-RCC cause death was increasingly important among RCC patients in recent two decades. Cardiovascular disease and other cancers were crucial co-morbidities that required significant attention in the management of RCC patients.

**Supplementary Information:**

The online version contains supplementary material available at 10.1186/s12889-023-15647-2.

## Introduction

Renal cell carcinoma (RCC) is a malignant tumor originating from the renal tubular epithelial cells, accounting for comprehensive 80% of all primary kidney tumors [1]. Worldwide, renal cell carcinoma is the sixth most common cancer in men and the tenth most common cancer in women [2]. A reported data suggested that North America had the highest worldwide estimated incidence of RCC, and the incidence of RCC continued to increase in the U.S. in recent decades [2, 3]. It may be partly due to the occasional increase in renal masses when abdominal imaging of nonspecific musculoskeletal or gastrointestinal diseases is performed [2]. In 2021, a total of 76,080 new cases of renal cell carcinoma (RCC) were diagnosed in the United States. RCC was a type of kidney cancer that has a relatively high mortality rate. According to a statistic, Uruguay had the highest estimated mortality rate of 4.4 per 100,000, followed by Argentina with 3.6 per 100,000, Chile with 3.1 per 100,000, and the USA with 2.6 per 100,000 [2, 4]. The risk factors reported included smoking, obesity, hypertension, and chronic kidney disease, and some potential risk factors incorporated environmental factors, co-morbidities, and analgesics ([2] http://www.cancerresearchuk.org/health-professional/cancer-statistics/statistics-by-cancer-type/kidney-cancer/incidence-heading-Zero).

Although the incidence rate of renal cell carcinoma has been increasing, some literature showed that the relative survival of RCC had improved significantly over the past three decades [5, 6]. For example, data from the SEER database presented the 5-years relative survival rate increased from 50% in 1975–1977 to 73% in 2003–2009 in the U.S. [3]. Meanwhile, a study that incorporated 32 European countries showed a decrease in mortality rate in some countries [6]. Considering that patients are relatively old at the time of diagnosis of RCC (high incidence of age 60–70 years) (http://www.cancerresearchuk.org/health-professional/cancer-statistics/statistics-by-cancer-type/kidney-cancer/incidence-heading-Zero), it is reasonable that patients often have co-morbidities. Therefore, it is likely that non-RCC causes of death will be increasingly more common among RCC patients in the coming years. By exploring the cause of death distribution of RCC patients, we can furtherly understand the role of these co-morbidities among RCC death cases to improve the management and survival of patients with RCC.

As a developed country, America's advanced medical technology and disease management strategy are often explored and used for reference by other countries. Up to our knowledge, there are still lacking enough studies exploring the distribution of causes of death in RCC patients. We hypothesized that although RCC is still the leading cause of death in patients with the disease, the proportion of people dying from non-RCC may be higher in recent years due to the development of the treatment [7–9]. In this study, the primary purpose is to explore the changing trend of the causes of death of RCC patients based on the information of causes of death obtained from the Surveillance, Epidemiology, and End Results (SEER) database (1992–2018). Meanwhile, further examination is also performed by stratifying the SEER population by age at diagnosis, sex, race, stage, and treatment.

## Materials and methods

### Study population and data sources

This study referred to a secondary analysis of the databases of the SEER Program of the National Cancer Institute, which had collected information on cancer incidence and mortality in the U.S. We obtained cases from the case list of *Incidence—SEER Research Plus Data, 13 Registries, Nov 2020 Sub (1992–2018),* covering approximately 13.4% of the U.S. population (based on 2010 census). List of ‘Site Recode ICD-O-3/WHO 2008 classification’ and ‘C.S. Schema—AJCC 6th Edition’ were used to select patients diagnosed with RCC. Meanwhile, we excluded patients with age < 15, unknown survival time, or unknown cause of death. We identified the following variables: age at diagnosis, sex, race, SEER historic stage, surgery information, chemotherapy recode, cause of death and survival month.

Death cases were obtained from the case listing of *Incidence-Based Mortality—SEER Research Plus Data, 13 Registries, Nov 2020 Sub (1992–2018)*. Different from traditional mortality, Incidence-based mortality (IBM) cases linked mortality records to incident cancer cases and can be calculated by variables like tumor stage, age, and gender.

### Cause of death data

The cause of death (COD) in the SEER database was corded with the International Classification of Diseases, Ninth Edition (ICD-9) from 1979 to 1998, and the ICD-10 was used to code patients who died after 1998. Based on the seer's classification of COD, we divided the cause of death of RCC patients into six common categories for facilitating analysis: renal cell carcinoma; other cancers (digestive system cancers, respiratory system cancers, urinary system cancers, leukemia and other cancers); cardiovascular diseases (diseases of heart, hypertension without heart disease, cerebrovascular diseases, atherosclerosis, other diseases of arteries, arterioles, capillaries); respiratory diseases (pneumonia and influenza, chronic obstructive pulmonary disease and Allied cond); other non-cancer diseases(Alzheimers, chronic liver disease and cirrhosis, diabetes mellitus, and other diseases); non-disease cause(accidents and adverse effects, homicide and legal intervention, and other cause of death).

### Statistical analysis

We described and compared the characteristics of patients who died of RCC and non-RCC. Two-sample t-tests were used for continuous variables and the Chi-square test for categorical variables. We evaluated the distribution of causes of death in RCC patients by calculating the percentage of total deaths of six kinds of COD and the cumulative incidence of death for each selected cause over survival time. In addition, further examination in COD distribution was stratified by age at diagnosis, sex, race, summary stage, surgery, and chemotherapy. Survival time was defined as the period of RCC diagnosis to the recorded date of death. We used SEER*Stat software (version 8.3.9) to calculate the annual age-standardized mortality rates (adjust to 2000 US Standard Population) and calculated by 100 000 person-years. The National Cancer Institute’s Joinpoint Regression Program (Version 4.6.0.0) was utilized to identify the best-fitting log-linear regression model to exhibit the trend of IBM rates [10]. Meanwhile, we also perform Joinpoint Regression to calculate annual percentage change (APC) and 95% confidence interval (CI) to quantify the mortality trends. Further examination was performed by age at diagnosis, sex, race, and summary stage. All data analysis was completed by SPSS 22.0 (BM Corp, Armonk, NY).

## Results

### Characteristics of the study population by cause of death

Finally, we enrolled 107,683 cases diagnosed with RCC from the SEER database. 25,376(23.6%) cases among them were confirmed death cause of RCC, and 27,178(25.34%) patients were recorded with non-RCC death (Table 1). The group of non-RCC death cases tended to have more localized stage tumors (68.3%vs21.2%, *P* < 0.001). In contrast, the proportion of cases with distant metastasis was significantly prominent in the group of patients who died of RCC (47.3%vs 7.9%, *P* < 0.001). Cases that received surgery were more common in the non-RCC death group (75.0% vs 54.6%, *P* < 0.001), while patients who received chemotherapy were more prominent in the group of patients who died of RCC (18.4% vs 2.8%, *P* < 0.001). The median and mean survival months in the non-RCC death group were significantly longer than those in the RCC death group (55 vs 12 for median survival; 72.706 vs 30.269 for mean survival, all *P* < 0.001) (Table 1).

**Table 1:** Distribution of causes of death for people diagnosed with renal cell carcinoma

Population characteristics	Overall patients No. (%)	Died from RCC No. (%)	Died from other cause No. (%)	*P*-value
**All patients**	107,683(100%)	25,376(100%)	27,178(100%)	
**Age(years)**				
Median age (25th–75th percentile)	62(52–72)	67.5(57.5–77.5)	72(62–77.5)	< 0.001
Mean age	63.42	65.971	69.598	0.003
**Sex**				< 0.001
Female	40,363(37.5%)	8715(34.3%)	9963(36.7%)	
Male	67,320(62.5%)	16,661(65.7%)	17,215(63.3%)	
**Race**				< 0.001
White	87,915(81.6%)	21,063(83.0%)	22,096(81.3)	
Black	10,124(9.4%)	2158(8.5%)	3228(11.9%)	
Others^a^	9644(9.0%)	2155(8.5%)	1854(6.8%)	
**Summary stage**				< 0.001
Localized	57,712(53.6%)	5376(21.2%)	18,560(68.3%)	
Regional	14,615(13.6%)	5247(20.7%)	3979(14.6%)	
Distant	15,002(13.9%)	12,003(47.3%)	2141(7.9%)	
Unknown	20,354(18.9%)	2750(10.8%)	2498(9.2%)	
**Undergoing surgery**				< 0.001
Yes	85,304(79.2%)	13,865(54.6%)	20,381(75.0%)	
No	21,778(20.2%)	11,330(44.6%)	6613(24.3%)	
Unknown	601(0.6%)	181(0.7%)	184(0.7%)	
**Chemotherapy**				< 0.001
Yes	6744(6.3%)	4659(18.4%)	774(2.8%)	
No	100,939(93.7%)	20,717(81.6%)	26,404(97.2%)	
**Cause of death**				
RCC	25,376(23.6%)	25,376(100%)	0(0%)	NA
Non-disease cause	4023(3.7%)	0(0%)	4023(14.8%)	
Other cancers	8003(7.4%)	0(0%)	8003(29.4%)	
Non-cancer disease	4195(3.9%)	0(0%)	4195(15.4%)	
Cardiovascular diseases	9023(8.4%)	0(0%)	9023(33.2%)	
Respiratory diseases	1934(1.8%)	0(0%)	1934(7.1%)	
Censored^a^	55,129(51.2%)	0(0%)	0(0%)	
**Median survival month** (25th–75th percentile)	50(14–113)	12 (3–39)	55(17–112)	< 0.001
**Mean survival month**	72.608	30.269	72.706	< 0.001

*RCC* renal cell carcinoma

*NA* not application

*P*-value: The *p*-value indicates whether there is a statistically significant difference in the distribution of variables between patients died from RCC and died from other cause

^a^ others include American/Indian/Alaska/Native and Asian/Pacific Islander

### Trends in the cause of death distribution of RCC patients

**Fig. 1:**
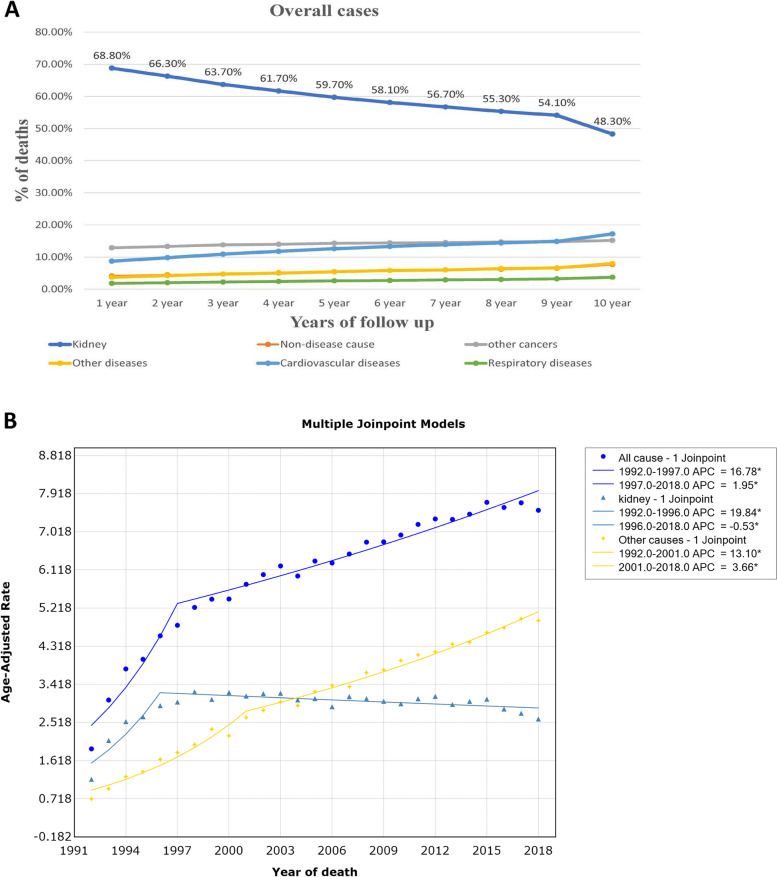
**A** Distribution of the most common causes of death in all renal cell carcinoma patients by survival time. **B** The estimated annual percentage change (APC) and 95% confidence intervals (CI) of mortality rates from Renal cell carcinoma using joinpoint regression

Among the study population, the leading cause of death among RCC cases was renal cell carcinoma, which accounted for 48.3% (25,376) of deaths. This was followed by cardiovascular diseases at 17.2% (9023), other cancers at 15.2% (8003), other non-cancer diseases at 8% (4195), non-disease causes at 7.7% (4023), and respiratory diseases at 3.6% (1934) (Table 1). Patients with RCC had a higher likelihood of dying from RCC than from other causes across all study populations (Fig. 1A). However, the proportion of patients who passed away from renal cell carcinoma gradually decreased with the progression of survival time. Meanwhile, the percentage of patients who succumbed to cardiovascular diseases, other cancers, other non-cancer diseases, non-disease causes, and respiratory diseases showed a slight increase over time. Moreover, after a follow-up period of more than nine years, the proportion of RCC patients dying from cardiovascular disease had even surpassed that of patients dying from other cancers.

We performed a subgroup analysis of the overall population by age, sex, race, tumour stage, and treatment. The cause of RCC death was the most common in all other subgroups except in RCC patients diagnosed with localized stage (Supplement Figs. 1, 2, 3, 4 and 5). Of RCC patients with localized stage, cardiovascular disease death cases accounted for a more significant proportion than those who died of RCC (26.4% vs 22.5%). For RCC patients of different age groups, the secondary causes of death were different. Other cancer was the secondary COD in RCC patients age < 50 years, 50–60 years, and 60–70 years (Supplement Fig. 1ABC), whereas the secondary COD in patients age > 70 years was cardiovascular diseases (Supplement Fig. 1D). The cardiovascular diseases were the secondary cause of death both in male and female RCC patients. In contrast, the proportion of patients who died of other cancers was higher than that of patients who died from cardiovascular disease in the early follow-up (before 8 years for female, before 9 years for male) (Supplement Fig. 2). The distribution of secondary causes of death did not differ in ethnic distribution among patients with renal cell carcinoma (Supplement Fig. 3). Of RCC patients with localized and regional stage, cardiovascular disease death cases had a higher proportion than other cancer death cases (26.4% vs 20.4% for localized; 15.2% vs 11.7% for regional) (Supplement Fig. 4AB). Other cancers were the secondary causes of death in patients with the distant stage (Supplement Fig. 4C). Cardiovascular diseases were the second leading cause of death after RCC in patients with RCC who had undergone surgery. Other cancer death cases were more common than those who died from cardiovascular diseases in patients without surgery (Supplement Fig. 5AB). Among all patients receiving chemotherapy, the proportion of patients who died from other cancers was significantly higher than that of cardiovascular disease death cases at each follow-up period. However, this result was only consistent with those without receiving chemotherapy in the early follow-up period (before 7 years) (Supplement Fig. 5CD).

Table 2 exhibited the changing trend in the proportion of RCC and non-RCC death cases during the study period. The proportion of RCC death cases present a continuous decrease with the rate of 69.71%, 59.03%,50.47%,45.05% and 38.96% for the calendar period of 1992–1996, 1997–2001, 2002–2006, 2007–2011 and 2012–2018, respectively. On the contrary, we observed a stable increase in the proportion of non-RCC death cases with the rate of 30.29%, 40.97%, 49.53%, 54.95% and 61.04% for the calendar period of 1992–1996, 1997–2001, 2002–2006, 2007–2011 and 2012–2018, respectively (Table 2).

**Table 2:** Trends in cause of death by calendar years (1992–2018)

Year of deaths	All cause death cases No. (%)	RCC death cases No. (%)	Other cause death cases No. (%)
1992–1996	5197(100%)	3623(69.71%)	1574(30.29%)
1997–2001	9153(100%)	5403(59.03%)	3750(40.97%)
2002–2006	11,478(100%)	5793(50.47%)	5685(49.53%)
2007–2011	14,093(100%)	6349(45.05%)	7744(54.95%)
2012–2018	24,879(100%)	9694(38.96%)	15,185(61.04%)

*RCC* renal cell carcinoma

### Trends in incidence-based mortality of renal cell carcinoma and other cause death

Of all-cause death cases, the incidence-based mortality of RCC showed an initial significant increase at a rate of 16.8% (95%CI: 11.9–21.9,* P* < 0.001) from 1992 to 1997, then began to decelerate with the APC of 2.0% (95%CI: 1.6–2.3, *P* < 0.001) after 1997 (Fig. 1B, Table 3). We observed a significant increase in IBM rate of RCC (APC:19.8%; 95%CI: 11.1–29.2; *P* < 0.001) for the period of 1992–1996 and then a slight downward trend at the rate of -0.5% (95%CI: -0.9–0.1; *P* = 0.009) among all RCC death cases (Fig. 1B, Table 3). For cases recorded with other cause deaths, the IBM rate of RCC exhibited a rapid increase during 1992–2001 (APC = 13.1%, 95%CI: 10.9–15.4; *P* < 0.001), and following a relatively slower increase with the APC of 3.7% (95%CI: 3.2–4.1, *P* < 0.001) (Fig. 1B, Table 3).

**Table 3:** The estimated annual percentage change (APC) and 95% confidence intervals (CI) of mortality rates by cause of deaths for different subgroup cases

Characteristics	All-cause			Died from RCC			Died from other cause		
	**Period**	**APC (95% CI)**	**P-value**	**Period**	**APC (95% CI)**	**P-value**	**Period**	**APC (95% CI)**	**P-value**
**Overall patients**	1992–1997	16.8% (11.9–21.9)	< 0.001	1992–1996	19.8% (11.1–29.2)	< 0.001	1992–2001	13.1% (10.9–15.4)	< 0.001
	1997–2018	2.0% (1.6–2.3)	< 0.001	1996–2018	-0.5% ( -0.9–0.1)	0.009	2001–2018	3.7% (3.2–4.1)	< 0.001
**Age**									
< 60	1992–1994	65.0% (28.6–111.6)	< 0.001	1992–1994	69.5% (25.1–129.7)	0.002	1992–2001	14.1% (9.6–18.8)	< 0.001
	1994–2018	2.3% (2.0–2.5)	< 0.001	1994–2018	-0.3% ( -0.70–1)	0.099	2001–2018	5.3% (4.5–6.0)	< 0.001
60–70	1992–1997	18.2% (12.4–24.3)	< 0.001	1992–1997	15.0% (7.9–22.6)	< 0.001	1992–2001	15.5% (12.2–18.8)	< 0.001
	1997–2018	2.6% (2.2–2.9)	< 0.001	1997–2018	-0.6% ( -1.1–0.01)	0.034	2001–2018	4.7% (4.1–5.3)	< 0.001
> 70	1992–1998	13.3% (9.2–17.6)	< 0.001	1992–1995	28.1% (13.0–45.3)	< 0.001	1992–1999	15.8% (12.1–19.7)	< 0.001
	1998–2018	1.5% (1.1–1.9)	< 0.001	1995–2018	-0.4% ( -0.8–0.01)	0.0501	1999–2018	3.0% (2.6–3.4)	< 0.001
**Sex**									
Male	1992–1997	17.1% (11.4–23.2)	< 0.001	1992–1996	21.0% (9.7–33.5)	0.001	1992–2002	11.4% (9.4–13.5)	< 0.001
	1997–2018	2.0% (1.6–2.3)	< 0.001	1996–2018	-0.3% ( -0.8–0.2)	0.180	2002–2018	3.3% (2.8–3.8)	< 0.001
Female	1992–1996	21.5% (13.3–30.4)	< 0.001	1992–1994	23.6% (16.2–31.4)	< 0.001	1992–1999	18.3% (13.1–23.7)	< 0.001
	1996–2018	1.8% (1.5–2.1)	< 0.001	1994–2018	-1.2% ( -1.4–1.0)	< 0.001	1999–2018	4.0% (3.5–4.5)	< 0.001
**Race**									
White	1992–1997	17.1% (12.0–22.4)	< 0.001	1992–1996	20.1% (11.4–29.6)	< 0.001	1992–2001	13.7% (11.5–15.9)	< 0.001
	1997–2018	2.1% (1.8–2.4)	< 0.001	1996–2018	-0.4% ( -0.8- -0.01)	0.039	2001–2018	3.9% (3.5–4.3)	< 0.001
Black	1992–1995	35.1% (15.5–58.1)	0.001	1992–1995	33.8% (9.4–63.6)	0.007	1992–1999	14.4% (7.7–21.6)	< 0.001
	1995–2018	2.0% (1.6–2.4)	< 0.001	1995–2018	-0.8% (-1.4–0.3)	0.007	1999–2018	3.2% (2.5–4.0)	< 0.001
**Stage**									
Localized	1992–2003	15.1% (11.7–18.5)	< 0.001	1992–2002	14.1% (8.4–20.0)	< 0.001	1992–2005	13.7% (11.3–16.1)	< 0.001
	2003–2018	2.8% (1.9–3.8)	< 0.001	2002–2018	-0.1% ( -1.5–1.3)	0.902	2005–2018	3.0% (2.0–4.1)	< 0.001
Regional	1992–1999	15.9% (8.1–24.2)	< 0.001	1992–1996	31.9% (7.7–61.4)	0.010	1992–2001	16.7% (11.2–22.4)	< 0.001
	1999–2018	-0.4% ( -1.4–0.6)	0.390	1996–2018	-1.4% ( -2.3–0.4)	0.008	2001–2018	0.9% ( -0.2–1.9)	0.097
Distant	1992–2015	-0.01% (-0.8- 0.8)	0.916	1992–2015	-0.2% ( -1.0–0.6)	0.589	1992–2015	-0.1% ( -0.6–0.5)	0.804
	2015–2018	-49.0% (-62.4–30.8)	< 0.001	2015–2018	-52.3% ( -66.3- -32.7)	< 0.001	2015–2018	-22.3% ( -29.6–14.2)	< 0.001

*RCC* renal cell carcinoma, *APC* annual percentage change, *CI* confidence interval

The non-RCC causes IBM rate had exceeded RCC-specific IBM rate after 2005 (Fig. 1B). Similar trends were observed in RCC patients aged < 60 years, 60–70 years and > 70 years (Supplement Fig. 6, Table 3). For RCC patients with regional stage, the trend in all-cause IBM rates exhibited a sharp increase at the rate of 15.9% (95%CI: 8.1–24.2; *P* < 0.001) before 1999, then slightly decreased with the APC of -0.4% thereafter (95%CI: -1.4–0.6) (Table 3). The RCC-specific IBM rate showed an initial prominent increase and then started decreasing at the rate of -1.38% (95%CI: -2.3–0.4) in 1996. Meanwhile, we observed a relatively rapid increase in non-RCC cause IBM before 2001, then beginning to slowly increase with APC of 0.9% (95%CI: -0.2–1.9) (Table 2). For RCC patients with distant stage, the IBM rates showed a similar slight decrease with the rate of -0.01% (95%CI:-0.8- 0.8), -0.2%(95%CI: -1.0–0.6), and -0.1%(95%CI: -0.6–0.5) for all-cause, RCC-specific, and non-RCC cause, respectively, from 1992 to 2015, then following a prominent decrease(APC = -49.0%, 95%CI: -62.4–30.8, *P* < 0.001 for all-cause, APC = -52.3%, 95%CI: -66.3- -32.7, *P* < 0.001 for RCC cause; APC = -22.3%, 95%CI: -29.6–14.2,* P* < 0.001 for non-RCC cause) (Table 3).

## Discussion

This study examined temporal trends in causes of death among patients with RCC over 27 years in the SEER population. We found that renal cell carcinoma is still the leading cause of death for RCC patients, despite improving the treatment and survival in patients with RCC. However, the proportion of patients who died of RCC has decreased significantly from 69.71% in 1992–1996 to 38.96% in 2012–2018. Meanwhile, the secondary causes of death were often different when we stratified the RCC patients. Notably, the RCC-specific mortality rate exhibited a slight decrease, but the non-RCC cause mortality rate stably increased among RCC patients in recent two decades.

The decrease in the proportion of death causes of RCC might be due to the improvement of treatment and frequent surveillance. In recent decades, significant progress has been made in treating localized and advanced RCC. For patients with localized RCC, competitive options for the treatment of these patients included radical or partial nephrectomy, thermal ablation or active surveillance, and a recent meta-analysis showed no significant differences in metastasis-free survival between two kinds of treatment over a mean follow-up of 47.1 months [11]. Despite advances in understanding the biology of renal cell carcinoma, surgery remains the primary treatment for RCC patients [5]. The prominent change was that more and more nephron-sparing surgery was performed since increasing evidence suggested no significant differences in cancer control were observed between radical nephrectomy and nephron-sparing surgery, especially for those with T1 stage tumours [5, 12]. This change also benefits from improving the surgical approach. Robot-assisted nephron-sparing surgery became more popular because challenging cases can be treated by minimally invasive surgery. A body of retrospective reports showed that the overall survival rate of patients treated with nephron-sparing surgery was improved compared with radical nephrectomy [13, 14]. In addition, compared with radical nephrectomy, nephron-sparing surgery had a long-term protective effect on the risk of postoperative cardiovascular events [15]. In this study, the cardiovascular disease had become the leading cause of death over RCC among cases with localized stage tumors. This result showed that the treatment of early RCC had worked well on cancer control, and some complications had become a more critical part of renal cell carcinoma management.

For patients with advanced RCC, the effect and utilization of surgery were relatively insufficient. Only 40% of patients with tumors recurrence were provided with surgical resection, and this value was significantly lower in patients with metastatic disease [1, 16]. The production of targeted therapy based on molecular level had changed the treatment mode of RCC patients, especially those with the advanced-stage tumor in recent ten years [1, 8]; However, due to the lack of enough substantial evidence to prove its effect and relatively low adoption rate, we cannot make an optimistic conclusion on the treatment effect of advanced RCC patients. This might be why we found that RCC still had a significant dominant proportion in the cause of death in patients with regional and distant stages. Notably, the mortality of RCC patients with regional and distant stages has shown a slightly decreasing trend in the recent 20 years. A pretty evident decline was presented in cases with the distant stage from 2015 to 2018. This might be due to more and more patients with RCC being found by accident with the expansion of routine imaging examination of many diseases. More patients were found and treated at an early stage.

Simultaneously, overtreatment should be noticed considering the increase in the incidental detection of renal masses [2]. A retrospective study based on the SEER data found the rates of renal surgery parallel the increasing incidence of kidney cancer from 1983 to 2002 [17]. However, the mortality rate has similarly continued to increase. This apparent disconnect—between increased treatment and increased mortality—suggested that the benefits of small kidney mass surgery need to be carefully evaluated. Interestingly, our study found a significant increase in mortality in patients with localized stage, in parallel with the increase in non-RCC cause mortality. However, there was no significant change in the RCC-specific mortality in the recent 20 years. Some renal function losses and surgical complications caused by excessive surgery, especially radical nephrectomy, might increase the non-RCC mortality. A body of studies has reported that radical nephrectomy significantly increases the risk of cardiovascular death for those with kidney masses [5, 11, 15, 17]. Meanwhile, RCC patients surgically treated tended to be long-term cancer survivors (85 – 96% cancer-specific survival 10 years after surgery) [18, 19]. Therefore, it was no surprise that non-RCC death became a more crucial part of the cause of death than RCC among patients with RCC.

Non-RCC cause of death included a wide range of diseases, with cardiovascular diseases and other cancers being the most significant. In the past decade, there have been some noticeable changes in the leading causes of death among general population patients in the United States. According to recent statistics, heart disease has surpassed cancer as the leading cause of death among cancer patients [20]. This indicated that the mortality distribution in renal cell carcinoma patients was similar to that of the general American population. However, it is worth noting that according to mortality rate data from the last decade, we observed a downward trend in both cardiovascular disease (APC = -4.1) and cancer mortality (APC = -2.3) in the general American population. Some studies have pointed out that suffering from cancer and cancer treatment might increase the risk of cardiovascular death [21, 22]. Although lacking similar literature indicating that RCC increased the risk of cardiovascular death, some studies had suggested that partial nephrectomy or radical nephrectomy might increase the risk of cardiovascular death [5, 11, 15, 23]. For those with early or low-stage RCC, cardiovascular disease should get more attention in RCC management when treatment for RCC achieves a relatively ideal effect. A study based on the Norway population enrolled 1425 RCC patients and examined the proportion of cases with two or more malignant tumors [24, 25]. They found a significantly higher risk of accompanying other subsequent malignancies in patients with RCC. Similar results had been reported in previous studies [26]. Therefore, it is not surprising that other cancers were also an important part of the cause of death in patients with renal cell cancer.

Some limitations of this study needed to be noted. Firstly, the causes of death were divided into six common categories in this study. Although this is conducive to the analysis and results, it also limits us from conducting more in-depth research to obtain more detailed information on the cause of death in RCC patients. In addition, we just focused on the primary causes of death. We ignored the contributing factors, which might overestimate or underestimate the number of deaths in RCC, as patients' deaths might be inappropriately attributed to their RCC. At the same time, we had to admit that there might be some coding errors of the cause of death in the SEER database considering such a piece of considerable population information. However, we expect that this will have a minimal impact on our study. We use the SEER stage to classify RCC patients to identify the cause of death distribution in patients with different distributions. Although this method was relatively simple, it might not be convenient for clinical application compared with the TNM system. Ultimately, the SEER database lacked some cancer information, such as smoking, detailed surgical methods, immunotherapy, targeted therapy and so on. However, we believe that the lack of this information will not affect the reliability of our results.

## Conclusion

Although the treatment of patients with RCC had made significant progress in recent decades, RCC was still the primary cause of death of patients with RCC, especially for those with advanced stage. However, non-RCC cause death played an increasingly important role in the cause of death of RCC patients in recent two decades. Cardiovascular disease and other cancers were crucial co-morbidities that required significant attention in the management of RCC patients. Meanwhile, in the future, more studies are needed to furtherly understand these co-morbidities to improve the management and survival of RCC patients and to make a more powerful explanation for these results.

## Supplementary Information

**Additional file 1: Supplement Figure 1.** Distribution of the most common causes of death in different age renal cell carcinoma patients by survival time.

**Additional file 2: Supplement Figure 2.** Distribution of the most common causes of death in different sex renal cell carcinoma patients by survival time.

**Additional file 3: Supplement Figure 3.** Distribution of the most common causes of death in different race renal cell carcinoma patients by survival time.

**Additional file 4: Supplement Figure 4.** Distribution of the most common causes of death in different stage renal cell carcinoma patients by survival time.

**Additional file 5: Supplement Figure 5.** Distribution of the most common causes of death in different renal cell carcinoma patients with treatment by survival time.

**Additional file 6: Supplement Figure 6.** The estimated annual percentage change (APC) and 95% confidence intervals (CI) of mortality rates in different age renal cell carcinoma patients.

## Data Availability

The data in this article comes from the SEER database This data can be found here: https://seer.cancer.gov/data-software/documentation/seerstat/nov2020/.
