# Supplementary material for: Trends in cause of death among patients with renal cell carcinoma in the United States: a SEER-based study
Source: BMC Public Health. 2023 Apr 26;23:770. doi: 10.1186/s12889-023-15647-2 (PMC10131378; doi:10.1186/s12889-023-15647-2)
Supplement: Supplementary file 3 — Additional file 3: Supplement Figure 3. Distribution of the most common causes of death in different race renal cell carcinoma patients by survival time. [file 12889_2023_15647_MOESM3_ESM.docx]

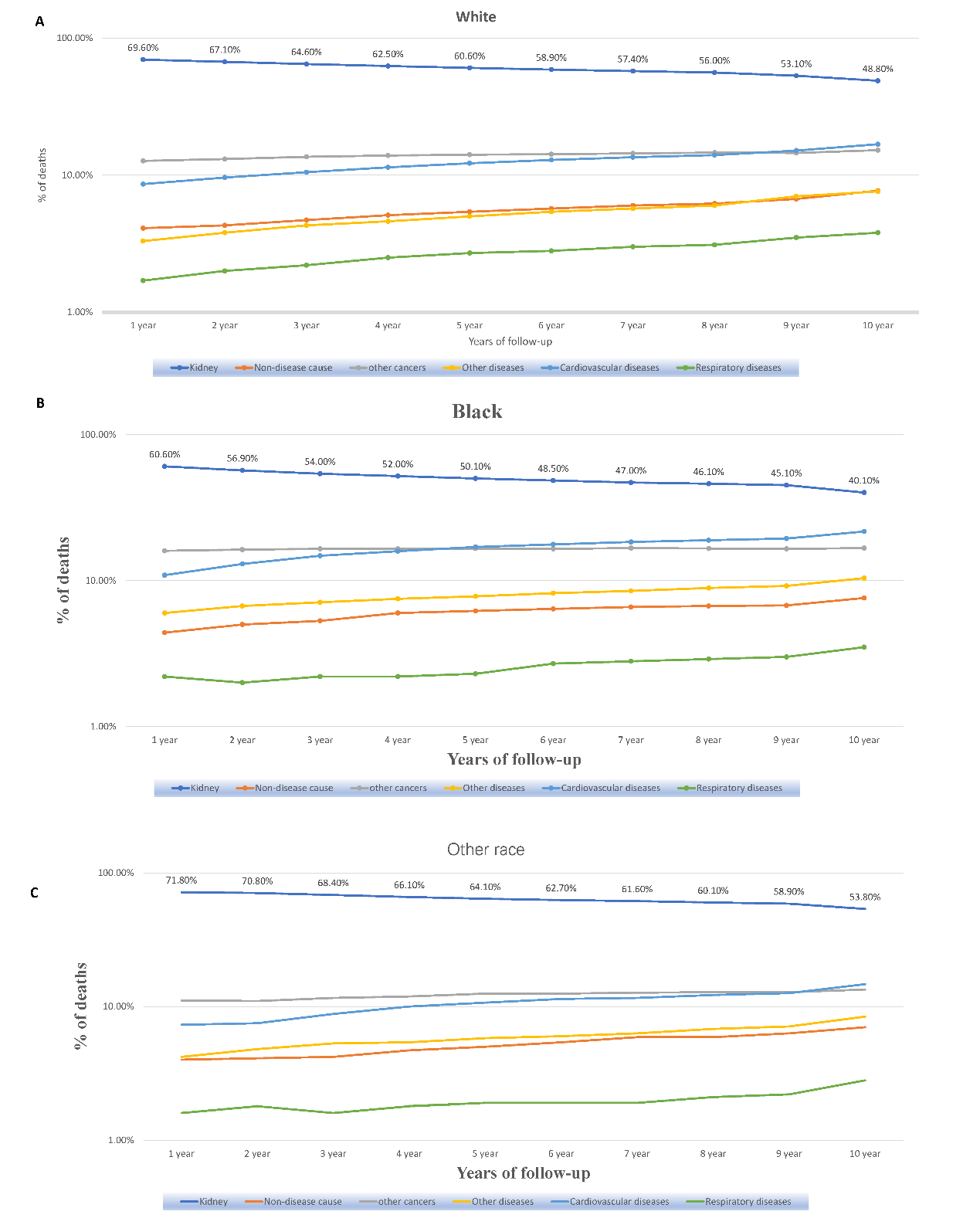


supplement Figure 3: Distribution of the most common causes of death in different race renal cell carcinoma patients by survival time
