# Supplementary material for: Trends in cause of death among patients with renal cell carcinoma in the United States: a SEER-based study
Source: BMC Public Health. 2023 Apr 26;23:770. doi: 10.1186/s12889-023-15647-2 (PMC10131378; doi:10.1186/s12889-023-15647-2)
Supplement: Supplementary file 6 — Additional file 6: Supplement Figure 6. The estimated annual percentage change (APC) and 95% confidence intervals (CI) of mortality rates in different age renal cell carcinoma patients. [file 12889_2023_15647_MOESM6_ESM.docx]

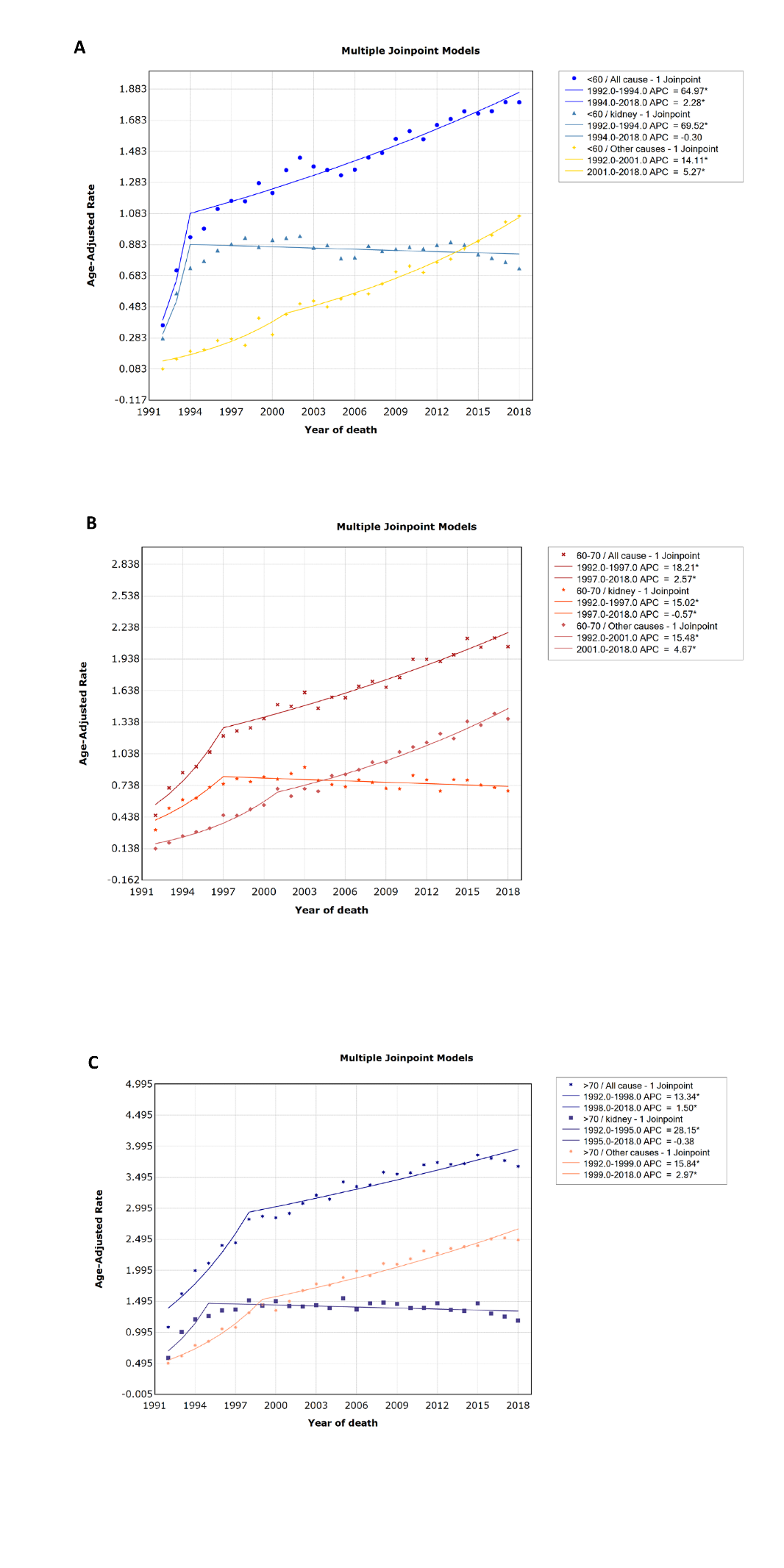


supplement Figure 6: The estimated annual percentage change (APC) and 95% confidence intervals (CI) of mortality rates in different age renal cell carcinoma patients
